# Implementing good life with OsteoArthritis from Denmark (GLA:D®) in public outpatient settings in Tasmania, Australia

**DOI:** 10.1016/j.ocarto.2026.100788

**Published:** 2026-03-27

**Authors:** Yoseph M. Alamneh, Laura Sutton, Christian Barton, Saliu Balogun, Katherine Lawler, Marina Djekanovic, Lisa J. O'Brien, Helen M. McDonald, Trena Youngblutt, Paula Hyland, Paul Harvie, Gregory M. Peterson, Natalie Collins, Barbara de Graaff, Pieter Van Dam, Graeme Jones, Dawn Aitken

**Affiliations:** a Menzies Institute for Medical Research, University of Tasmania, Hobart, Australia; b La Trobe University, Melbourne, Australia; c Tasmanian Health Service (THS), Hobart, Australia; d Royal Hobart Hospital Orthopaedic Department, Hobart, Australia; e School of Pharmacy and Pharmacology, University of Tasmania, Hobart, Australia; f School of Health and Rehabilitation Sciences: Physiotherapy, The University of Queensland, Brisbane, Australia; g School of Nursing, University of Tasmania, Hobart, Australia

**Keywords:** Education, Exercise, Implementation, Osteoarthritis

## Abstract

**Objective:**

Good Life with OsteoArthritis in Denmark (GLA:D®) is an evidence-based education and exercise program for people with hip or knee osteoarthritis. The program largely operates in private healthcare settings around the world. This study evaluated its implementation and delivery in public outpatient settings in Tasmania, Australia.

**Design:**

A process and outcome evaluation was conducted to evaluate the implementation and delivery of GLA:D® within the publicly funded Tasmanian Health Service. The evaluation was conducted using the RE-AIM QuEST Framework, including health system and service-level metrics, patient-level data and program fidelity. Semi-structured focus groups and qualitative interviews were conducted with staff and patients who participated in the program, with thematic analysis of the outputs.

**Results:**

**Implementation and Adoption**: GLA:D® was implemented at three outpatient clinics. Successful implementation factors included GLA:D® being a recognisable evidence-based program that was straightforward to deliver, executive support and buy-in, local physiotherapist champions, and the establishment of an eReferral pathway which improved efficiency. **Reach**: Twelve physiotherapists were trained in GLA:D®, while 89 patients enrolled in the program (21 cohorts), of which 63 participated in the GLA:D® registry at baseline. **Effectiveness**: Most patients noted improvements in symptoms; however, some patients with co-morbidities or complex pain did not benefit. **Maintenance**. All three centres have elected to continue the program.

**Conclusions:**

GLA:D® can be effectively, efficiently and sustainably implemented in public outpatient settings. Operational and administrative considerations have been identified and provide a foundation for broader implementation of the program in public and regional settings.

## 1 Introduction

Osteoarthritis (OA) is a highly prevalent, painful, disabling, and costly condition that affects over 595 million people worldwide [1]. OA has an unsustainably high economic burden; for example, in Australia the healthcare costs for OA were estimated to be AUD$4.3 billion annually [2]. The majority of healthcare costs associated with OA are related to knee and hip joint replacement surgeries [3].

Current guidelines for OA recommend patient education, exercise, and weight management (where indicated) as first-line treatment [[4], [5], [6]]. Surgery is only recommended when non-surgical treatments have been exhausted [4,5,7]. Despite this, many people with OA do not receive high-value, first-line care, even in the early stages of the condition, and there is an over-reliance on medications and surgery to treat the condition [8,9].

Good Life with OsteoArthritis in Denmark (GLA:D®) is a well-recognised surgical avoidance model of care, with evidence showing it may prevent or delay the need for joint replacement surgery [10,11]. It is a guideline-based, structured education and exercise program for patients with knee or hip OA [12] which has been implemented in 10 countries, including Australia [11]. While GLA:D® has been implemented across private and public settings [13], uptake has been predominantly in private clinics and metropolitan areas [11,12,14], with limited evaluation in public outpatient settings in Australia [11,13]. Private physiotherapy clinics provide services with fees charged to patients, and some consultations may be partially refundable via government rebates or private health insurance, but this is limited for group programs. Providing GLA:D® in a public outpatient service expands access with no out-of-pocket cost to the patient. Implementing GLA:D® in a public outpatient setting presents a new opportunity for providing high quality care at low cost.

Tasmania is an island state of Australia with a high burden of OA due to an ageing population, socioeconomic disadvantage and high obesity rates [15,16]. Tasmania has a population of approximately 576,000, distributed across inner-regional, outer-regional, and remote areas [17]. While most Australians (71%) live in major cities, Tasmania has no area classified as a major city [18]. Around two-thirds of Tasmanians live in inner-regional locations and one-third live in outer regional, remote or very remote locations. This presents unique health service provision challenges. Similar to other settings [11,19], our previous research in Tasmania has demonstrated that ‘out-of-pocket’ cost and accessibility to allied health professionals is a major barrier to accessing evidence-based care for OA [20,21]. To help increase access to high quality OA care for Tasmanians, we partnered with the Tasmanian Health Service (THS) to implement GLA:D® in public outpatient settings. The THS is a publicly funded healthcare service which operates hospitals and outpatient services around the state. The THS is managed by the Tasmanian Department of Health and funded by the Tasmanian government. The aim of this study was to evaluate the implementation and delivery of GLA:D® in public outpatient settings in Tasmania, Australia.

## 2 Methods

### 2.1 Study design and setting

Leaders within the THS Physiotherapy Department (LOB, HMD, TY), in collaboration with researchers, were responsible for setting up GLA:D® at the community outpatient clinics. An implementation strategy was developed to assess and ensure system readiness and select suitable clinics. Two clinics were initially selected based on practical requirements including space/room availability, equipment requirements, workforce availability, volume of patients and physiotherapist interest to undertake GLA:D® training. GLA:D® was initially launched in May 2022 at the two clinics and after 12 months was expanded to a third clinic due to increased demand and successful integration within the THS model. The number of physiotherapists working across the clinics varied, with some working across multiple clinics. The study was conducted from May 2022 to February 2024.

A local champion (MD), who was a junior physiotherapist, was responsible for managing the project on the ground and developing administrative pathways and resources for staff, including developing a standardised GLA:D® referral system for all clinics. Implementation involved coordinating GLA:D® training for physiotherapy staff, allocating resources and equipment, establishing referral processes, infrastructure, and data collection procedures, and disseminating information within the THS physiotherapy department.

The evaluation used a multiple-method design including qualitative and quantitative approaches. This research was conducted in accordance with the Declaration of Helsinki and was approved by the Tasmanian Human Research Ethics Committee (HREC 26867). This project received Tasmanian Department of Health Research Governance approval. All health professionals and patients provided written informed consent for face-to-face interviews and verbal consent for phone interviews prior to the commencement of the interview. This paper was prepared in line with the Standards for Reporting Implementation Studies and Consolidated Criteria for Reporting Qualitative Research checklists (Supplementary file 1) [[22], [23], [24]].

### 2.2 Process and outcome evaluation

**Fig. 1:**
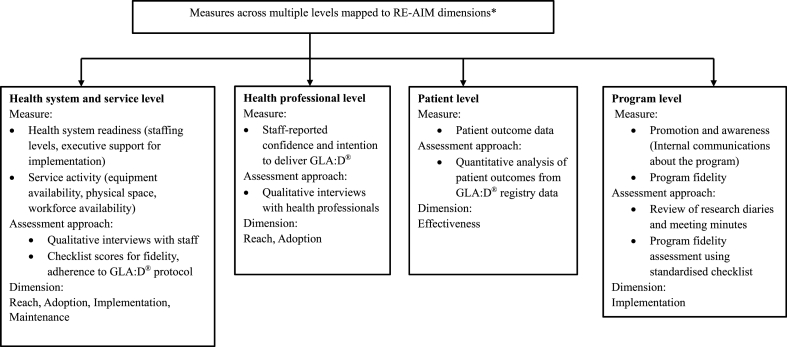
Measures across multiple levels mapped to the RE-AIM framework. ∗RE-AIM dimension: **Reach**: Proportion of participants and representativeness of individuals who participated in the program; **Effectiveness**: Intervention effects on patients' outcomes; **Adoption**: Number of participants and delivery of the program; **Implementation**: The extent to which the program was implemented by the staff members; **Maintenance**: The extent to which the program becomes part of routine THS practices and maintain effectiveness.

A process and outcome evaluation was conducted to evaluate the implementation and delivery of GLA:D® within the THS. The RE-AIM QuEST Framework was used to assess Reach, Effectiveness, Adoption, Implementation, Maintenance, and Qualitative Evaluation of Systematic Translation [25] using health system, service-level, and patient-level data and program fidelity (Fig. 1), consistent with a previous Australian program evaluation of GLA:D® [11].

### 2.3 Reach

#### 2.3.1 Patients

Patients referred to the THS Physiotherapy Department with knee and/or hip joint-related problems were eligible to participate in GLA:D® if they were: (i) aged 18 years and older; (ii) assessed by a physiotherapist to have knee and/or hip OA and (iii) deemed appropriate for non-surgical management. Eligible patients were offered a physiotherapy assessment to determine suitability and interest in attending the GLA:D® program. Those suitable were placed on a waiting list for GLA:D®, while others were returned to the waiting list for individual physiotherapy management.

### 2.4 Effectiveness

#### 2.4.1 GLA:D® intervention and data collection

GLA:D® has three components [13]: (i) two structured group education sessions; (ii) 12 1-h group exercise classes run over a six-week period; and (iii) collection of outcome data in the GLA:D® Australia registry [11]. Patients were encouraged to complete the registry questionnaires online. If patients expressed difficulty with the questionnaires at baseline, they were supported by staff to complete the questionnaire either online or using a paper-based version which was then uploaded by staff. We used patient-level data from the GLA:D® registry, including physical function tests (40 m fast-paced walk, 30 s chair stand) [13], 12-Item Knee injury and Osteoarthritis Outcome Score (KOOS-12) [26], 12-Item Hip disability and Osteoarthritis Outcome Score (HOOS-12) [27], pain severity over the past month [28], and EuroQoL five-dimension five-level quality of life measure (EQ-5D-5L) [29].

### 2.5 Adoption

Health system and service metrics were recorded at each site, including number of patients in the cohort, weekly session counts, number of staff and their role, and education format (face-to-face or telehealth). The number of patients who participated in GLA:D® were recorded as those who gave consent to participate in GLA:D® and booked into their first session. Patient attendance at the education and exercise sessions was recorded by physiotherapists, and home exercises were recommended to replace any missed group exercise sessions.

### 2.6 Implementation

Program fidelity was evaluated through direct observations of clinic operations, physical space, staffing resources, and program delivery. Observations were guided by a standardized checklist adapted from Davis 2018 [30], covering: (i) clinic operations and planning; (ii) environmental factors; (iii) clinician-related factors; (iv) GLA:D® Australia program-related factors; (v) patient factors; and (vi) education factors (Supplementary file 2). Process and outcome evaluation were kept by staff to document daily activities, observations, and reflections for program improvement.

#### 2.6.1 Qualitative data collection

Qualitative data was collected between August and December 2023. A grounded theory methodological approach was used in data collection and analysis. Semi-structured interviews were guided by interview schedules which were developed by the research team (including health professionals) after a review of the literature (Supplementary file 3). Interview guides allowed flexibility to accommodate the interviewee's experience [31], while ensuring that key themes were explored and were tailored to accommodate the different roles of staff members. Interviews were conducted either one-on-one, or in focus groups and either in person (at Menzies Institute for Medical Research or THS locations) or over the phone, by two experienced interviewers (LS, PhD and/or DA, PhD) with a trained student researcher (YA).

All staff involved in the implementation of GLA:D® within the THS were invited to participate in an interview, and all agreed (physiotherapists, allied health assistants, managers, and administrators).

Patients were eligible for interview if they had attended the THS GLA:D® program between May 2022 to December 2023. Patients were recruited through letters sent in two rounds and forms distributed during GLA:D® classes, using an opt-in approach. We included patients who had varying levels of completion and success with the program. Although efforts were made to balance sex, age, and attendance distribution, the final sample was based on convenience and availability of those who responded. Patients had no prior relationship with interviewers and were advised of the interviewers’ current position/role in the study.

Age, sex, residential postcode and centre where GLA:D® was completed was collected from all patients, along with centre location and years of practice from health professionals. Field notes were recorded during/after each interview and saved for reference during analysis. Patients could request transcripts for review, none did.

### 2.7 Data analysis

Quantitative data are presented as frequencies and percentages for categorical data and means and standard deviations for continuous data. Paired t-tests were used to assess mean difference between baseline and three-month follow-up scores. Effect size was calculated as the change in mean score from baseline to three months divided by the baseline standard deviation [32,33] and interpreted as small (≥0.20), moderate (≥0.50), or large (≥0.80) [34].

A responder analysis was also performed on the baseline and 3-month data. Patients were considered responders if they had improvement equal to or exceeding the minimal clinically important difference in at least one of the following measures at 3 months: KOOS/HOOS-QoL (15 mm [35]); average pain severity (15 mm [36]); worst pain severity (15 mm [36]); EQ-5D-5L index (0.07 [37]).

All interviews were audio recorded, transcribed verbatim by a third-party transcription service (SmartDocs) and de-identified. Two researchers (YA and LS) reviewed all transcripts and developed a coding framework using inductive thematic analysis [38]. All transcripts were coded in NVivo (Version 14, QSR International Pty Ltd, 2023). After developing the initial coding framework, YA and LS independently coded the interviews and reconvened to discuss coding decisions. Coding was refined through multiple iterations until a consensus was reached on the final codes. Iterative thematic analysis was used to generate the main themes from the primary codes, which were mapped to the RE-AIM framework [25]. The themes and sub-themes were discussed with members of the research team (YA, LS, DA, SB) and reviewed based on discussion. Analysis was reflexive [39] and involved discussion with the implementation team where clarification was required. Transcripts were coded and analysed while interviews were being conducted, the decision to cease interviewing was based on the team agreeing that data saturation had been reached as no new themes were emerging. Qualitative findings were presented as themes and subthemes and supported by quotes from staff and patients.

## 3 Results

### 3.1 Reach

Between May 2022 and February 2024, 12 physiotherapists were trained to deliver the program. Over 22 months, 89 patients enrolled in the program (21 cohorts). They ranged from 31 to 88 years of age (mean: 67.5, standard deviations 9.2), 84% (n = 53) were female, and 63 completed some or all of the GLA:D® registry questionnaire at baseline. Table 1 presents patient characteristics. Most patients (71%, n = 45) were overweight or obese, with a mean body mass index of 30.7 kg/m^2^ (SD = 8.3 kg/m^2^). The majority (79%, n = 50) of patients reported the knee as their primary joint affected by OA, and 13 patients reported hip OA.

**Table 1:** Patient characteristics.

Characteristics	Number of observations	n (%), unless otherwise stated
Age (years), mean [range]	89	67.5 [31–88]
Body mass index (kg/m^2^), mean [range]	58	30.7 [18.3–53.4]
Female sex	89	75 (84.3)
Australian born	63	44 (69.8)
Aboriginal or Torres strait Islander background	63	1 (1.6)
Marital status:	63	
Single		5 (7.9)
Married/domestic partnership/de facto		35 (55.6)
Widowed		6 (9.5)
Divorced/Separated		16 (25.4)
Prefer not to say		1 (1.6)
Highest education level achieved:	63	
Primary school		2 (3.2)
High school		21 (33.9)
Apprenticeship		2 (3.2)
Certificate		12 (19.4)
Diploma		4 (6.5)
Undergraduate degree		12 (19.4)
Postgraduate degree		9 (14.5)
Employment status:	60	
Unemployed		22 (36.7)
Part-time		6 (10.0)
Full-time		5 (8.3)
Home duties		5 (8.3)
Studying full-time		4 (6.7)
Retired		18 (30.0)
Primary joint affected by osteoarthritis:	63	
Knee		50 (79.4)
Hip		13 (20.6)

### 3.2 Qualitative findings

A total of 14 staff and 13 patients participated in the interviews (Fig. 3). The main themes were (1) acceptability of GLA:D. (to providers and patients); (2) patient experience and accessibility; (3) implementation challenges; and (4) sustainability within the THS®. The qualitative findings and themes are integrated throughout the results section, quotes are identified as staff or patient, with gender (woman or man) and affected joint (knee or hip).

### 3.3 Effectiveness outcomes

Table 2 presents the functional and patient-reported outcomes over 3 months. Of 89 patients who enrolled in GLA:D®, 58 attended their 3-month follow-up appointment and had functional assessments recorded. There were improvements in the 40 m walk test (mean difference 0.3; 95% CI 0.1–0.7 m/s) and the 30-s chair stand test (2.4; 95% CI 0.8–5.6 counts).

**Table 2:** Functional tests and patient-reported outcomes at baseline and 3 months and estimated mean differences and effect size between timepoints.

Outcomes	Baseline		3 months		Mean difference (95% CI)	Effect size (95% CI)	*P*-value
	n	Mean (SD)	n	Mean (SD)			
Functional measures							
40 m walk, m/s	89	1.2 (0.5)	58	1.5 (0.2)	0.3 (0.1–0.7)	0.7 (0.2–1.5)	**0.043**^a^
30s chair stand, count	89	11.6 (4.7)	58	13.5 (6.8)	2.4 (0.8–5.6)	0.4 (0.5–1.2)	**0.054**
Knee measures							
KOOS-12 (0–100)	49	50.6 (16.9)	15	63.3 (20.2)	12.5 (3.9–21.0)	0.7 (0.1–1.3)	**0.008**^a^
KOOS-12 pain (0–100)	49	48.9 (18.3)	15	64.2 (20.5)	14.3 (2.4–26.2)	0.8 (0.2–1.4)	**0.011**^a^
KOOS-12 function (0–100)	49	61.5 (19.6)	15	71.3 (21.5)	10.6 (1.9–19.2)	0.5 (0.1–1.1)	**0.010**^a^
KOOS-QoL (0–100)	49	41.5 (16.9)	15	54.6 (23.3)	12.5 (0.9–24.1)	0.7 (0.1–1.3)	**0.018**^a^
Hip measures							
HOOS-12 (0–100)	13	59.1 (14.5)	3	65.3 (37.1)	13.4 (104.3–131.2)	0.3 (1.0–1.6)	0.336
HOOS-12 pain (0–100)	13	53.0 (14.6)	3	66.7(38.2)	20.1 (96.5–136.8)	0.7 (0.6–2.0)	0.268
HOOS-12 function (0–100)	13	67.5 (18.33)	3	72.9 (36.6)	13.9 (99.7–127.4)	0.3 (1.0–1.5)	0.326
HOOS-QoL (0–100) s	13	56.7 (14.5)	3	56.3 (37.5)	6.3 (120.8–133.3)	0.0 (1.2–1.3)	0.852
Knee and hip measures combined							
Worst pain VAS (0–100)	62	61.9 (21.2)	17	58.2 (30.7)	−4.1 (−13.4–5.3)	0.2 (0.4–0.7)	0.368
Average pain VAS (0–100)	61	46.0 (21.6)	17	35.8 (23.6)	−4.2 (−13.4–5.0)	0.5 (0.1–1.0)	0.343
EQ-5D-5L VAS (0–100)	62	64.5 (22.0)	18	75.7 (14.2)	14.6 (2.8–26.4)	0.6 (0.0–1.1)	**0.019**^a^
EQ-5D-5L index (0–1)	63	0.7 (0.2)	18	0.8 (0.2)	0.0(-0.1–0.1)	0.25 (0.3–0.8)	0.841

n, number of observations at each time point; SD, standard deviation; CI, confidence interval; m/s, metres per second; KOOS, Knee injury and Osteoarthritis Outcome Score; HOOS, Hip disability and Osteoarthritis Outcome Score; QoL, quality of life; VAS, visual analogue scale; EQ-5D-5L, European Quality of Life-5 dimensions-5 levels.

a Significant at 0.05.

**Fig. 2:**
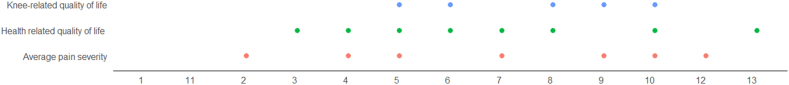
Responder analysis for 13 patients with complete baseline and 3-month data. Each dot indicates a patient who was a responder on a specific outcome measure at 3 months. X-axis indicates patient ID. Responsiveness was calculated as the number of responders divided by the number of patients with complete data.

A small proportion of patients completed the 3-month questionnaires (20%, n = 18). Of those 3-month questionnaires, 85% (n = 11) reported minimal clinically important difference in at least one of the outcome measures (pain intensity, KOOS-QoL or EQ-5D-5L) (Fig. 2).

**Fig. 3:**
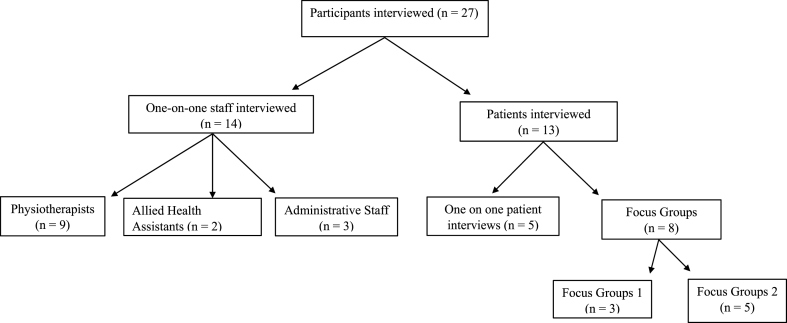
Flow diagram of participants who participated in a qualitative interview.

Most participants interviewed noted an improvement in symptoms. For example, one patient described the program as effective, “*it worked beautifully. After I started the program, it's sorted out [the pain] and I hardly ever have any pain anymore.”* (Patient 4, woman, knee). However, some patients with co-morbidities did not report benefits from the program (Table 3).

**Table 3:** Acceptability of the implementation and delivery of GLA:D® within public outpatient clinics.

Theme	Sub-theme	Illustrated Quotes
Acceptability of GLA:D® (to providers and patients)	GLA:D® being a recognisable evidence-based program	*“I think the other benefit may be actually from a service point of view … it's improved or made sure that I guess us as physios are providing the latest evidence in regard to the management.”* (physiotherapist 2, woman) *“It's an evidence based conservative management approach to helping people to live with osteoarthritis of their knee or hip. I really like evidence-based things, and I like things that save the health budget and avoid surgery”* (physiotherapist 8, woman)
	Program easy to deliver and can be individualised	*“The exercises that we're doing in the program are quite easy to deliver, they're simple movements, easy to demonstrate.”* (physiotherapist 7, woman)
	Education sessions do not allow flexibility	*“I don't know whether that was designed on purpose to have that personal interpretation attached to it or whether we wanted that to be a bit more standardised. I mean the information in the presentation's very standardised but your interpretation, the way to deliver and things sort of like I don't know whether there was a specific way to do it, etc.”* (physiotherapist 5, woman)
	Patients with co-morbidities or complex pain did not benefit	*“I didn't find it actually alleviated any of the pain for me … To fix one, I was hurting the other.”* (Patient focus group 1, woman, knee) *“We tend to find that if they had more co-morbidities …, they will really struggle.”* (physiotherapist, man 1 and AHA 1, woman) *“I do find the amount of exercise and the intensity of the exercise is quite a lot for patients, they'd generally be from their 60s, older, and possibly have other comorbidities.”* (physiotherapist 1, man)
	Group environment enhances patient motivation and adherence to the program	*“I think obviously the socialisation part of any group is often a benefit for people. So, they sort of motivate each other and whether that also leads to some connections and other opportunities that might arise that people sort of get to know other community activities that are out there that maybe some of the participants are doing and then they become interested in what they're doing”.* (Physiotherapist 4, man) *“… the motivation and that group interaction as much instead of doing it on your own. It's a bit boring on your own. But there was a group of I think 10 there this morning and we're doing it. And you know, it was friendly, and I said, “This is good.” Even though it's only the second time I've been to that group, great.”* (Patient focus groups 1, woman, hip)
	Accessibility	*“… if you are looking at people who may not have enough money for their own car or for transport and stuff, all of those sorts of factors can come into play to start the program.”* (Patient 3, woman, knee) *“… other thing I think that's been a bit challenging is people aren't necessarily willing to travel out of their area to attend a program … for some people the cost of travel, et cetera, can be a bit prohibitive or once again the times, they can't access public transport to get there, that type of thing as well.”* (physiotherapist 2, woman)
Effectiveness	Difficulty completing GLA:D® questionnaires	*“I found a few of the questions I thought were, I'm not quite sure what I need to answer here. It was a bit unclear for me but that happens with every survey that you do”.* (Patient focus groups 2, woman, knee)

Low completion of the GLA:D® three-month questionnaire prompted us to seek further feedback from patients and staff. If required, patients received assistance from staff to complete the baseline questionnaires, including assistance with reading, explanation of the questions, and having paper copies printed and then uploaded online by staff. The same assistance was not feasible for the 3-month questionnaires. Physiotherapists attributed the low completion to *“low literacy levels, limited IT access, poor IT literacy and questionnaire length”.* The font size of the questionnaire was also difficult for some patients. One physiotherapist said “*the questionnaires are so confusing … they're so wordy, they're so long … To decode that it takes so long.”* Patients also described difficulty with interpreting some of the questions (Table 3).

### 3.4 Adoption

#### 3.4.1 Patient adherence to the program

Ninety-six percent (n = 85) of patients attended at least one education session, and 82% (n = 70) attended both sessions. Adherence to the exercise sessions was high; out of 12 sessions, 79% (n = 70) of patients attended ≥6 sessions, with 47% (n = 42) attending 6–9, and 31% (n = 28) attending 10–12.

Patients and physiotherapists reported several reasons for non-adherence to exercise sessions, including comorbidities and access barriers such as exercise difficulty, travel, and costs.

### 3.5 Acceptability of GLA:D®

Sub-themes and supportive quotes are outlined in Table 3. GLA:D® was described positively by physiotherapists as an evidence-based program that was easy to implement and deliver in the THS. Physiotherapists liked that it was a recognisable brand and was straightforward to deliver. Whilst physiotherapists appreciated the content of the education sessions, some described the structure and flow as rigid, e.g.: *“Although I like the education sessions, I find they don't flow well for the way that I would present them*.*”* (Physiotherapist 2, woman).

The exercise components of the program were described by physiotherapists as simple to demonstrate, modify, and progress. Physiotherapists described the program as easily individualised for patients with varying strength and fitness levels, while still being simple to deliver as a group exercise class. However, physiotherapists acknowledged that some older patients and those with comorbidities struggled and may be better suited to one-on-one support (Table 3). Physiotherapists also noted benefits of the group format, including patient motivation and adherence. Patients expressed enjoying the group classes*: “I'm not great at doing exercises at home … it's way easier when I'm with other people.”* (Patient 3, woman, knee).

Patients enjoyed having free access to the program in their local area, and clinic location was a significant consideration in enrolling in GLA:D®. Considerations included distance from home, availability of public transport, transportation costs and availability of parking. *“I think for me that it was local was very helpful. If I'd had to travel to town, I probably wouldn't have done it. I would have found it a lot harder. But being local, knowing the area, knowing the parking, that made it*.” (Patient Focus group 2, woman, knee).

### 3.6 Implementation and sustainability considerations

Implementation and sustainability sub-themes and quotes are outlined in Table 4 and included: staffing and GLA:D® certification (including staff turnover and sick leave cover), administrative load, scheduling, and physical space.

**Table 4:** Implementation and sustainability considerations for the delivery of GLA:D® within public outpatient clinics.

Theme	Sub-theme	Illustrated Quotes
Implementation and sustainability considerations	Staffing and GLA:D® certification	*“The GLA:D® certification to begin with was very rigid, I guess, like these are the dates that's available; this is the time that you have to be there … it was a bit difficult to try and balance work and GLA:D®.”* (physiotherapist 5, woman). *“We gave people the option to become GLA:D® -trained, and not all people wanted to do that.” “… some people felt it was an additional thing that they have to do. So, there was that interest in - getting GLA:D® -trained was the first challenge.”* (physiotherapist 3, woman). *“If we keep increasing the locations of the groups then we probably would have to increase the percent of our staff that are trained”* (physiotherapist 8, woman)
	Staff leave and turnover of trained staff	*“… people will leave, so you need to be thinking about succession planning and training people and ensuring that there's cover when people are on leave and so on.”* (administrative 3, man) *“Well, staff leave, staff sick leave …. then there's no one to pick up their class. That's been a bit of a challenge … that's more difficult, with the centres being dispersed.”* (physiotherapist 3, woman) *“… when trained staff go on leave, leave the service, are unwell, that ability to be agile enough to be able to fill those gaps … that's [a] challenge.”* (physiotherapist 2, woman)
	Administrative load, scheduling, physical space	*“… it takes quite a lot of time in terms of set up and pack down, there's a reasonably heavy admin load for GLA:D®”.* (Physiotherapist 8, woman) *“At the beginning we had lots of issues, for example, three-month reviews– it was hard to book them in because after three months they're like, “Oh, I don't think I can” or [other] excuses. Always the booking in was hard, sometimes they don't answer the phone calls”.* (Physiotherapist 1, man and AHA 1, woman) *“Organising the equipment that was being used- you have to book the gym space. So, it's often booked by other exercise classes, so then you're restricted in time.”* (physiotherapist 3, woman)
Successful implementation factors	Leadership support and buy-in	*“… from my perspective, I'm certainly supportive of it … it's been supported from higher levels within allied health by the executive director of allied health.* *The support from UTAS was welcome.”* (administrative 3, man) *“I have a lot of support. I have administrative support; I have [Team leader], who is the coordinator.”* (physiotherapist 6, woman)
	Champions within the service	*“When we started to implement into the service, [a local champion-MD] did a couple of presentations and contacted the GP liaison so the word - I think people are quite familiar with it now within the department and within THS.”* (physiotherapist 3, woman) *“… there has been a few TV things that were done and also a newspaper article that [a local champion-MD] was involved in.”* (physiotherapist 2, woman)

To implement the program within the THS as an evaluation project, a hospital ethics and governance application was required. This was a difficult process to navigate, particularly for clinicians with less research experience. It also contributed to major delays in commencing GLA:D® (17 months).

Patients were encouraged to attend at least one education session before commencing the exercise sessions but sometimes sessions were cancelled due to staff sickness. The option of a rolling program was considered, but not feasible due to staff availability. Centres briefly trialled a hybrid education session via zoom, which was not adopted long-term. Maintaining participation and attendance at education, exercise, and follow-up sessions was challenging. “*I have had people who show up for the education sessions, one or two GLA:D*® *[exercise classes] and then disappear*.” (Physiotherapist 6, woman). Another physiotherapist noted, “*It's been challenging to get people to participate for their three-month outcome measures [appointment].*” (Physiotherapist 2, woman).

#### 3.6.1 Staffing and GLA:D® certification

Most staff who were GLA:D® certified were trained as part of this project. Some physiotherapists found the training difficult to balance with their workload, others did not see the need to become GLA:D® certified, feeling they had sufficient experience in OA treatment. Long-term staffing and maintaining an adequate number of GLA:D® trained physiotherapists to cover leave and accommodate staff rotation and turnover was identified as a challenge.

#### 3.6.2 Administrative load, scheduling and physical space

Scheduling the three-month review appointment was identified as a challenge. Patients often hesitated to commit to appointments, providing various reasons including uncertainty about their availability. The process of contacting patients was also time-consuming and required administrative support. Booking gym space and equipment was a barrier/challenge, as these spaces were often reserved for other exercise classes, restricting available times for program sessions (Table 4).

#### 3.6.3 Successful implementation factors

Executive support, physiotherapist champions, and the establishment of an electronic referral (eReferral) pathway facilitated implementation. *“implementation guide is very clearly written. [A local champion-MD] did a fantastic job. It was really helpful. It does outline admin and what I'm responsible for; and then, what the allied health assistants and so on are responsible for as well.”* (Admin 1, woman). The eReferral pathway improved the number of referrals coming to the program and communication between staff members.

To ensure the GLA:D® classes operated at full capacity (6–8 patients per class), knee and hip OA were prioritised for assessment on the clinic waitlist. This resulted in reduced wait times for knee and hip OA patients at the clinics.

### 3.7 Maintenance

The program has been effectively integrated into routine practices within the THS and all three centres have continued to offer the program.

### 3.8 Fidelity of GLA:D® program

Fidelity checks occurred on 2 occasions and showed that the program was delivered as recommended by GLA:D® Australia.

## 4 Discussion

This study evaluates implementation and delivery of GLA:D® in a regional Australian public health setting. With the exception of adherence to registry data collection, GLA:D® was effectively and sustainably implemented. So far, GLA:D® has limited implementation in public settings in Australia [13]. This study describes key implementation factors specific to delivery in a public setting.

Effective promotion within the department and executive support were key facilitators of program setup, uptake, and sustainability. A physiotherapist champion was crucial in developing processes (including referral) and promoting the program. Our findings were consistent with a previous Australia-wide evaluation [11], which identified central program support, opportunities for further professional development, and service providers’ positive attitudes toward program adoption as key enablers of service delivery.

The GLA:D® data registry is a core component of the program, requiring patients to complete online questionnaires; however, several challenges were identified. The baseline completion rate was 71%, comparable to findings from previous evaluations in Australia (75%) [11] and Canada (69%) [40]. However, only 20% (18/89) of patients completed the three-month follow-up compared to 54% (1044/1945) [11] in the national GLA:D® Australia registry and 57% (554/974) [40] in GLA:D® Canada. Collins et al. reported a 71% (50/70) three-month follow-up completion rate in a public hospital setting [41], however a 6-week follow-up was included in the study to ensure completion, compared to the pragmatic approach of our study.

Low literacy and limited access to technology were identified by clinicians as key barriers to the registry data collection. This was particularly noticeable at three-month follow-up, when time and resources were limited and physiotherapists/allied health assistants were unable to assist with questionnaire completion as they did for baseline. To improve accessibility, we recommend simplifying the questionnaire language and shortening the length. Having clinicians supporting patients to complete questionnaires may also help improve response rates but increases staff burden.

Attendance at the education sessions was similar to a previous study in an Australian public hospital setting (82% vs 79%) [41]. However, adherence rates for exercise sessions were lower, with only 31% of patients attending 10–12 sessions compared to 70% in the previous study [41]. Clinic location, limited public transport, and transportation costs were identified as barriers to patient adherence. These findings are consistent with other studies evaluating barriers to uptake of the program [19,42]. Telehealth and GLA:D® delivery via video conferencing may be a potential opportunity to address transport- and cost-related barriers.

Some patients with comorbidities did not benefit from the program and may have benefited from a more individualised approach. Previous GLA:D® data has demonstrated that patients with comorbidities have similar outcomes to patients without [43]. Therefore, the observed difference in our study is likely due to the small sample size. Previous evidence has not demonstrated a consistent association between patient characteristics (e.g., demographics, BMI, comorbidities, disease severity) and outcomes from first-line interventions such as exercise therapy and education [44,45]. However, a recent study by Peiris et al. reported that metabolic syndrome risk factors (e.g., obesity) were associated with outcomes following first-line care [46]. These findings suggest that more research is required into how patients with comorbidities respond to exercise interventions. There may be potential to develop improved screening procedures to predict who might benefit from the program.

Staffing, resourcing, and implementation support from leadership were identified as key sustainability factors for ongoing program implementation and delivery. These findings align with previous studies highlighting that organizational support and adequate staffing are critical for successful implementation of healthcare interventions [47]. We did not conduct an economic evaluation but note that the project was supported by research funding, which covered the cost of GLA:D® training and supported an increase in staffing, which could be a barrier to wider implementation in public services without this support.

While this study presents a high-quality multiple method evaluation of GLA:D® in a public setting, there are some limitations. First, only 20% of patients completed registry data collection at 3 months, limiting conclusions on short-term effectiveness in this population and context. Second, there is potential for response bias from patients who had a more positive experience, however, some patients reported they did not benefit from the program in qualitative interviews and non-responders are represented in quantitative data.

A strength of this study is the use of purposive sampling to recruit all staff involved in the delivery of GLA:D® in the THS. This approach captured all perspectives from executive level to administration support. The process evaluation allowed for data collection and feedback in real-time. GLA:D® was successfully implemented and has been maintained across three outpatient clinics.

## 5 Conclusion

Implementation of GLA:D® within Tasmanian public outpatient settings was demonstrated to be feasible, deliverable and acceptable to patients and providers. Factors for successful implementation have been identified and provide a foundation for broader and sustainable program implementation in public settings. Low questionnaire completion rates highlight the need for additional approaches that are acceptable to the target population. Successful implementation was supported by the program's evidence-based structure, executive support, local physiotherapy champions, and the establishment of an eReferral pathway.

## Author contributions

All authors contributed to the drafting and revision of the manuscript and approved the final version. Yoseph Alamneh (yosephmerkeb.alamneh@utas.edu.au) and Dr Laura Sutton (laura.sutton@utas.edu.au) ensures the integrity of the entire work.

**Conception and design:** Dawn Aitken, Christian Barton, Katherine Lawler, Yoseph Alamneh, Laura Sutton, Saliu Balogun, Marina Djekanovic, Lisa J O'Brien, Helen M McDonald, Trena Youngblutt, Paula Hyland, Gregory Peterson, Natalie Collins, Barbara de Graaff, Pieter Van Dam, Paul Harvie, Graeme Jones.

**Acquisition of data:** Yoseph Alamneh, Laura Sutton, Christian Barton, Saliu Balogun, Katherine Lawler, Marina Djekanovic, Lisa J O'Brien, Helen M McDonald, Trena Youngblutt, Dawn Aitken.

**Analysis:** Yoseph Alamneh, Laura Sutton, Saliu Balogun, Dawn Aitken.

**Interpretation of the data:** All authors.

**Drafting of the manuscript:** Yoseph Alamneh, Laura Sutton, Saliu Balogun, Dawn Aitken.

**Critical review of the manuscript for important intellectual content:** All authors.

**Obtained funding:** Dawn Aitken, Katherine Lawler, Paul Harvie, Lisa J O'Brien, Christian Barton, Paula Hyland, Barbara de Graaff, Pieter Van Dam, Gregory Peterson, Natalie Collins, Graeme Jones.

## Ethical considerations

This research was conducted in accordance with the Declaration of Helsinki and was approved by the Tasmanian Human Research Ethics Committee (HREC 26867). All health professionals and patients provided written informed consent for face-to-face interviews and verbal consent for phone interviews prior to the commencement of the interview.

## Data availability statement

The data generated from this study will not be deposited in a public repository due to privacy and consent restrictions. De-identified data can be made available from the corresponding author on reasonable request, subject to a data sharing agreement.

## Funding/support and role of the funding source

This study was funded by a Royal Hobart Hospital Research Foundation Project Grant. Funding and support was also provided by the Tasmanian Collaboration for Health Improvement. Royal Hobart Hospital Research Foundation and Tasmanian Collaboration for Health Improvement had no role in the study design, collection, analysis, and interpretation of data; in the writing of the manuscript; and in the decision to submit the manuscript for publication.

## Declaration of competing interest

Yoseph Alamneh: nothing to disclose.

Laura Sutton: nothing to disclose.

Christian Barton: Is a project lead for the implementation of GLA:D® Australia, a not-for-profit initiative which trains health professionals to provide guideline-based education and exercise therapy to people with osteoarthritis.

Saliu Balogun:

Katherine Lawler: Was a manager in the THS Physiotherapy Department during the commencement of the study.

Marina Djekanovic: nothing to disclose.

Lisa J O'Brien: nothing to disclose.

Helen M McDonald: nothing to disclose.

Trena Youngblutt: nothing to disclose.

Paula Hyland: nothing to disclose.

Paul Harvie: nothing to disclose.

Gregory Peterson: nothing to disclose.

Natalie Collins: nothing to disclose.

Barbara de Graaff: nothing to disclose.

Pieter Van Dam: nothing to disclose.

Graeme Jones: nothing to disclose.

Dawn Aitken: NHMRC/Medical Research Future Fund fellowship.
