# Supplementary material for: Implementing good life with OsteoArthritis from Denmark (GLA:D®) in public outpatient settings in Tasmania, Australia
Source: Osteoarthr Cartil Open. 2026 Mar 27;8(2):100788. doi: 10.1016/j.ocarto.2026.100788 (PMC13091989; doi:10.1016/j.ocarto.2026.100788)
Supplement: Multimedia component 1 [file mmc1.docx]

**Supplementary file 1**

| **eTable 1**: Consolidated criteria for reporting qualitative studies (COREQ) checklist | | |  |
| --- | --- | --- | --- |
| Topic | | Guide questions/description | Reported on page/ Y/N |
| Domain 1: Research team and reﬂexivity | | |  |
| Personal Characteristics | | |  |
| 1 | Interviewer/facilitator | Which author/s conducted the interview or focus group? | 6 |
| 2 | Credentials | What were the researcher’s credentials? | 6 |
| 3 | Occupation | What was their occupation at the time of the study? | 6 |
| 4 | Gender | Was the researcher male or female? | - |
| 5 | Experience and training | What experience or training did the researcher have? | 6 |
| *Relationship with participants* | | |  |
| 6 | Relationship established | Was a relationship established prior to study commencement? | 6 |
| 7 | Participant knowledge of the interviewer | What did the participants know about the researcher? | 6 |
| 8 | Interviewer characteristics | What characteristics were reported about the interviewer/facilitator? | 6 |
| Domain 2: Study design | | |  |
| *Theoretical framework* | | |  |
| 9 | Methodological orientation and Theory | What methodological orientation was stated to underpin the study? | 6 |
| Participant selection | | |  |
| 10 | Sampling | How were participants selected? e.g. purposive, convenience, consecutive | 6 |
| 11 | Method of approach | How were participants approached? e.g. face-to-face, telephone, email | 6 |
| 12 | Sample size | How many participants were in the study? | 8 |
| 13 | Non-participation | How many people refused to participate or dropped out? Reasons? | 6 |
| *Setting* | | |  |
| 14 | Setting of data collection | Where was the data collected? e.g. home, clinic, workplace | 6 |
| 15 | Presence of non-participants | Was anyone else present besides the participants and researchers? | 6 |
| 16 | Description of sample | What are the important characteristics of the sample? e.g. demographic data, date | 8 |
| Data collection | | |  |
| 17 | Interview guide | Were questions, prompts, guides provided by the authors? Was it pilot tested? | 6 |
| 18 | Repeat interviews | Were repeat interviews carried out? If yes, how many? | 8 |
| 19 | Audio/visual recording | Did the research use audio or visual recording to collect the data? | 6 |
| 20 | Field notes | Were ﬁeld notes made during and/or after the interview or focus group? | 6 |
| 21 | Duration | What was the duration of the interviews or focus group? | - |
| 22 | Data saturation | Was data saturation discussed? | 7 |
| 23 | Transcripts returned | Were transcripts returned to participants for comment and/or correction? | 6 |
| Domain 3: Analysis and ﬁndings | | |  |
| Data analysis | | |  |
| 24 | Number of data coders | How many data coders coded the data? | 7 |
| 25 | Description of the coding tree | Did authors provide a description of the coding tree? | Y |
| 26 | Derivation of themes | Were themes identiﬁed in advance or derived from the data? | 7 |
| 27 | Software | What software, if applicable, was used to manage the data? | 7 |
| 28 | Participant checking | Did participants provide feedback on the ﬁndings? | - |
| Reporting | | |  |
| 29 | Quotations presented | Were participant quotations presented to illustrate the themes / ﬁndings? Was each quotation identiﬁed? e.g. participant number | Throughout, tables |
| 30 | Data and ﬁndings consistent | Was there consistency between the data presented and the ﬁndings? | Y |
| 31 | Clarity of major themes | Were major themes clearly presented in the ﬁndings? | Y |
| 32 | Clarity of minor themes | Is there a description of diverse cases or discussion of minor themes? | Y |


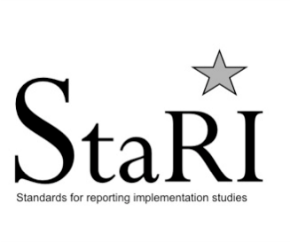
**Standards for Reporting Implementation Studies: the StaRI checklist for completion**

The StaRI standard should be referenced as: Pinnock H, Barwick M, Carpenter C, Eldridge S, Grandes G, Griffiths CJ, Rycroft-Malone J, Meissner P, Murray E, Patel A, Sheikh A, Taylor SJC for the StaRI Group. Standards for Reporting Implementation Studies [(StaRI) statement](http://www.bmj.com/content/356/bmj.i6795.full). *BMJ* 2017;356:i6795

The detailed Explanation and Elaboration document, which provides the rationale and exemplar text for all these items is: Pinnock H, Barwick M, Carpenter C, Eldridge S, Grandes G, Griffiths C, Rycroft-Malone J, Meissner P, Murray E, Patel A, Sheikh A, Taylor S, for the StaRI group. Standards for Reporting Implementation Studies [(StaRI). Explanation and Elaboration document](http://bmjopen.bmj.com/content/7/4/e013318.full?ijkey=vv4LKZxc25YcLJv&keytype=ref). *BMJ Open* 2017 2017;7:e013318

Notes: A key concept of the StaRI standards is the dual strands of describing, on the one hand, the implementation strategy and, on the other, the clinical, healthcare, or public health intervention that is being implemented. These strands are represented as two columns in the checklist.

| The primary focus of implementation science is the implementation strategy (column 1) and the expectation is that this will always be completed. | The evidence about the impact of the intervention on the targeted population should always be considered (column 2) and either health outcomes reported or robust evidence cited to support a known beneficial effect of the intervention on the health of individuals or populations. |
| --- | --- |

The StaRI standardsrefers to the broad range of study designs employed in implementation science. Authors should refer to other reporting standards for advice on reporting specific methodological features. Conversely, whilst all items are worthy of consideration, not all items will be applicable to, or feasible within every study.

| **eTable 2**: Standards for Reporting Implementation Studies (StaRI) statement checklist | | | | | |
| --- | --- | --- | --- | --- | --- |
| Checklist item | | Reported on page/ Y/N | Implementation Strategy | Reported on page/ Y/N | Intervention |
|  | |  | “Implementation strategy” refers to how the intervention was implemented |  | “Intervention” refers to the healthcare or public health intervention that is being implemented. |
| Title and abstract | | | | | |
| 1 | Title | 1 | Identification as an implementation study, and description of the methodology in the title and/or keywords | | |
| 2 | Abstract | 2 | Identification as an implementation study, including a description of the implementation strategy to be tested, the evidence-based intervention being implemented, and defining the key implementation and health outcomes. | | |
| Introduction | | | | | |
| 3 | Introduction | 3 | Description of the problem, challenge or deficiency in healthcare or public health that the intervention being implemented aims to address. | | |
| 4 | Rationale | 5 | The scientific background and rationale for the implementation strategy (including any underpinning theory/framework/model, how it is expected to achieve its effects and any pilot work). | 5 | The scientific background and rationale for the intervention being implemented (including evidence about its effectiveness and how it is expected to achieve its effects). |
| 5 | Aims and objectives | 3 | The aims of the study, differentiating between implementation objectives and any intervention objectives. | | |
| Methods: description | | | | | |
| 6 | Design | 4 | The design and key features of the evaluation, (cross referencing to any appropriate methodology reporting standards) and any changes to study protocol, with reasons | | |
| 7 | Context | 4 | The context in which the intervention was implemented. (Consider social, economic, policy, healthcare, organisational barriers and facilitators that might influence implementation elsewhere). | | |
| 8 | Targeted ‘sites’ | 4 | The characteristics of the targeted ‘site(s)’ (e.g locations/personnel/resources etc.) for implementation and any eligibility criteria. | 4 | The population targeted by the intervention and any eligibility criteria. |
| 9 | Description | 5 | A description of the implementation strategy | 5 | A description of the intervention |
| 10 | Sub-groups | - | Any sub-groups recruited for additional research tasks, and/or nested studies are described | | |
| Methods: evaluation | | | | | |
| 11 | Outcomes | - | Defined pre-specified primary and other outcome(s) of the implementation strategy, and how they were assessed. Document any pre-determined targets | 5 | Defined pre-specified primary and other outcome(s) of the intervention (if assessed), and how they were assessed. Document any pre-determined targets |
| 12 | Process evaluation | 5 | Process evaluation objectives and outcomes related to the mechanism by which the strategy is expected to work | | |
| 13 | Economic evaluation | - | Methods for resource use, costs, economic outcomes and analysis for the implementation strategy | - | Methods for resource use, costs, economic outcomes and analysis for the intervention |
| 14 | Sample size | 6 | Rationale for sample sizes (including sample size calculations, budgetary constraints, practical considerations, data saturation, as appropriate) | | |
| 15 | Analysis | 7 | Methods of analysis (with reasons for that choice) | | |
| 16 | Sub-group analyses | - | Any a priori sub-group analyses (e.g. between different sites in a multicentre study, different clinical or demographic populations), and sub-groups recruited to specific nested research tasks | | |

| Results | | | | | |
| --- | --- | --- | --- | --- | --- |
| 17 | Characteristics | - | Proportion recruited and characteristics of the recipient population for the implementation strategy | 8 | Proportion recruited and characteristics (if appropriate) of the recipient population for the intervention |
| 18 | Outcomes | - | Primary and other outcome(s) of the implementation strategy | 9 | Primary and other outcome(s) of the Intervention (if assessed) |
| 19 | Process outcomes | 8-12 | Process data related to the implementation strategy mapped to the mechanism by which the strategy is expected to work | | |
| 20 | Economic evaluation | 11 | Resource use, costs, economic outcomes and analysis for the implementation strategy | 9 | Resource use, costs, economic outcomes and analysis for the intervention |
| 21 | Sub-group analyses | - | Representativeness and outcomes of subgroups including those recruited to specific research tasks | | |
| 22 | Fidelity/ adaptation | 12 | Fidelity to implementation strategy as planned and adaptation to suit context and preferences | - | Fidelity to delivering the core components of intervention (where measured) |
| 23 | Contextual changes | 11 | Contextual changes (if any) which may have affected outcomes | | |
| 24 | Harms | - | All-important harms or unintended effects in each group | | |
| Discussion | | | | | |
| 25 | Structured discussion | 13 | Summary of findings, strengths and limitations, comparisons with other studies, conclusions and implications | | |
| 26 | Implications | 13 | Discussion of policy, practice and/or research implications of the implementation strategy (specifically including scalability) | 13 | Discussion of policy, practice and/or research implications of the intervention (specifically including sustainability) |
| General | | | | | |
| 27 | Statements | 17, 18 | Include statement(s) on regulatory approvals (including, as appropriate, ethical approval, confidential use of routine data, governance approval), trial/study registration (availability of protocol), funding and conflicts of interest | | |

**Supplementary file 2**

| **eTable 3.** Program Fidelity checklist | | | |
| --- | --- | --- | --- |
| Factors | Criterion | Status* | Comments |
| Clinic Operations and Planning | Does the clinic have a GLA:D® protocol (e.g., booking procedures)? | ☐ Not Met / ☐ Partially Met / ☐ Completely Met |  |
| Environmental | Adequate space: Participants visible to trainers, tripping hazards addressed. | ☐ Not Met / ☐ Partially Met / ☐ Completely Met |  |
|  | Appropriate exercise equipment (e.g., sliders, balance stick). | ☐ Not Met / ☐ Partially Met / ☐ Completely Met |  |
| Clinician-Related | Instructions: Clear, appropriate, ensures participant understanding. | ☐ Not Met / ☐ Partially Met / ☐ Completely Met |  |
|  | Feedback: Provides adequate feedback and allows participant questions. | ☐ Not Met / ☐ Partially Met / ☐ Completely Met |  |
|  | Exercise: Prescribes suitable exercises and progression. | ☐ Not Met / ☐ Partially Met / ☐ Completely Met |  |
|  | Professionalism: Starts on time, dresses appropriately, interacts professionally. | ☐ Not Met / ☐ Partially Met / ☐ Completely Met |  |
| Program-related | All components included: Warm-up, posture alignment, core stability, etc. | ☐ Not Met / ☐ Partially Met / ☐ Completely Met |  |
|  | Pain monitoring: During and post-session. | ☐ Not Met / ☐ Partially Met / ☐ Completely Met |  |
|  | Rate of Perceived Exertion (RPE): Participants encouraged to report. | ☐ Not Met / ☐ Partially Met / ☐ Completely Met |  |
| Participant | Demonstrates understanding of therapist instructions. | ☐ Not Met / ☐ Partially Met / ☐ Completely Met |  |
|  | Accepts direction/feedback from therapists. | ☐ Not Met / ☐ Partially Met / ☐ Completely Met |  |
|  | All participants are engaged in the exercises. | ☐ Not Met / ☐ Partially Met / ☐ Completely Met |  |
| Education | Allocates adequate time per slide. | ☐ Not Met / ☐ Partially Met / ☐ Completely Met |  |
|  | Speaks clearly, uses understandable language, and explains jargon. | ☐ Not Met / ☐ Partially Met / ☐ Completely Met |  |
|  | Answers questions accurately or makes time to follow up if unsure. | ☐ Not Met / ☐ Partially Met / ☐ Completely Met |  |
|  | Time allocated for session (mins). | ☐ Not Met / ☐ Partially Met / ☐ Completely Met |  |

***** Not Met: Deficits affecting multiple participants or creating safety issues that require immediate attention. Partially Met: Deficits affecting a single participant or minor issues requiring improvement. Completely Met: No deficits affecting program quality or safety.

**Supplementary file 3**

| **eTable 4.** Interview script for osteoarthritis patients**.** Questions are indicated for each topic. The choice of prompt will vary from interview to interview depending on how information flows. | |
| --- | --- |
| Topic | Question/Prompt |
| Interaction with the GLA:D® program | How did you hear about the GLA:D® program?  How did you come to participate in the GLA:D® program?  Which centre did you attend the program?  Did you complete the whole program? If not, was there anything specific that made it difficult for you to complete the program? |
| The GLA:D® experience | Can you tell me about your experience with the GLA:D® program?  What did you like about the program?  Was the program what you expected it to be?  Did it help with your knee/hip osteoarthritis?  Did you find the education sessions useful?  Was there anything you did not like about the program?  Was it in a location that was easy for you to access?  Were any of the programs delivered via Telehealth, and if so, how did you find this? |
| Impact on osteoarthritis management and outlook | Has it changed how you manage your knee/hip osteoarthritis (Prompts: change in medication, change in activity level, plans for surgery)?  Will you continue the exercises you learnt in the program?  Have you continued the exercises you learnt in the program?  If yes- what motivates you to continue the exercises?  If not- why not? Anything that would make it easier or more difficult to continue the exercises? |
| Previous management of osteoarthritis | What types of health professionals have you previously seen for your knee/hip osteoarthritis?  Have you had any knee or hip surgery for your osteoarthritis? And if so, what was done and why?  Apart from surgery, can you tell me about any other treatments you have received for your knee/hip osteoarthritis?  Can you tell me about any education you’ve received from your health professionals about your knee/hip osteoarthritis? (Prompts: face-to-face education, flyer, booklet, online information etc.)  Have you previously been given exercise for your knee/hip osteoarthritis? If so, by who? (Prompts: specific professions.) If so, what did it involve? (Prompts: supervision, number of sessions per week/day, how long each session, how many weeks, etc.)  How do you think the exercises affect your knee/hip osteoarthritis? |
| Barriers and enablers to starting the program | Can you tell me about anything that you would consider when deciding whether or not to start the GLA:D® program?  How important to you is the advice or recommendation of your doctor or specialist when considering whether or not to start the GLA:D® program?  Is there anything that would make it easier for you or for other patients to start the GLA:D® program?  Is there anything that would make it more difficult for you or for other patients to start the GLA:D® program? |
| Promotion of the program and accessibility | Would you recommend the program to a friend or family member?  Do you think the THS should continue to offer the program?  Would you be willing to pay for the program? |
| Basic demographic data:  How old are you?  Male/female (by observation)  What is the postal code of your residential address?  Outpatient clinic where you received GLA:D®?  Is there anything further you would like to tell me about any of the things we’ve talked about today?  (Thanks, and good-bye) | |

| **eTable 5.** Interview script for health professionals. Questions are indicated for each topic. The choice of prompt will vary from interview to interview depending on how information flows. | |
| --- | --- |
| Topic | Question/Prompt |
| Interaction with the GLA:D® program | Can you please describe your role in the THS?  What role do you play in relation to the THS GLA:D® program? (Ask for more details if unclear) |
| Knowledge about the program | What is your understanding of the GLA:D® program?  What is your understanding of the qualifications and experience of the staff who run the GLA:D® program? |
| Health Professionals delivering the GLA:D® program | Were you previously GLA:D® trained or were you trained for the THS program? Were you motivated to become GLA:D® trained? What motivated you to become GLA:D® trained?  How would you describe the level of difficulty in delivering the program in the THS setting?  What were some of the challenges delivering the program within the THS?  Was anything about delivering the program easier than you expected?  Was any part of the program delivered via Telehealth, and if so, how did you find this?  How did you previously manage OA before the implementation of the GLA:D® program? |
| The GLA:D® experience | What do you see as some of the benefits of the program?  Do you think the program has been implemented successfully?  What are some barriers to its implementation and sustainability**?**  What would the program require to continue to run in the THS?  Are there any aspects of the GLA:D® program you liked? If so, please elaborate?  Are there any aspects of the GLA:D® program you disliked? If so, please elaborate? |
| Referring Health Professionals: | Can you tell me about your experience referring patients to the GLA:D® program?  Was the referral process efficient?  Are they any factors that would make it easier to refer into the program? (Prompts include broad options: referral process, program effectiveness, confidence in intervention and intervention givers, cost vs benefit, access issues for patient, patient preference.)  Did you feel confident referring patients to the program?  Will you continue to refer patients to the program if it is offered within the THS?  Was there good awareness of the program in the department you work in?  Describe any difficulties you have encountered (or potentially would encounter) when/if referring your patient to the GLA:D® program?  Do you have anything else you would like to add about the management of knee/hip osteoarthritis? |
| Promotion of the program | Do you think there was good promotion/awareness of the program? Would you promote the program more widely to the general public or just to GPs?  Would you recommend the program to a friend or family member?  Do you think the THS should continue to offer the program? |
| Basic demographic data:  How old are you?  Male/female (by observation)  Years of practice:  Main location of work:  Is there anything further you would like to tell me about any of the things we’ve talked about today?  (Thanks, and good-bye) | |

| eTable 6: Codes/themes | |
| --- | --- |
| Name | Description |
| Barriers and Challenges | Any mention of a barrier or challenge or anything that was particularly difficult or impacted on implementation and delivery of the program. Barriers and/or challenges that are specifically related to the daughter nodes are coded there. |
| Logistics | References to the logistics of running GLA:D^®^, including managing space, purchasing equipment, managing patients, processes, booking patients, contacting patients. |
| Patient Barriers | Staff and patients perceived or observed barriers that patients encounter that impact on their ability to access or participate in the GLA:D^®^ program in the THS. This may include distance to the centre, travel cost or accessibility, co-morbidities that prevent participation, disease severity, and commitment to the program and literacy including reading and writing ability, computer literacy, English as a second language or non-English speakers having difficulty with access. |
| Staff | Any non-specific barriers related to staff or staffing that impact delivery or implementation of the program. |
| Training | Motivation for staff to be trained, complexities about training, staff response to training, other staff training references to be coded here. |
| Workload | References to staff workload here, balancing regular duties with GLA:D^®^, how GLA:D^®^ impacts on their workload and the workload of running the program. Administrative load for GLA:D^®^ is also coded here. References to frequent/regular staff turnover and the difficulties associated with a transient staff. References to staff leaving, difficulties retaining staff and how this impacts the implementation and delivery of GLA:D^®^. |
| Delivery of GLA:D® | Comments about delivering the program- staff leading the program, any aspects about leading the program or comments about delivering GLA:D^®^. Timing of the classes, opening hours of the centres, mention of classes running during working hours, any reference to time being a barrier or challenge for class access for patients but also for staff running the classes. And days that the classes are run on. |
| Dislikes about the program | Aspects about GLA:D^®^ that staff and patients do not like or do not enjoy including things that they would modify or change. |
| Implementation | Any references about participants' perceptions/reactions on whether the program was successfully implemented. |
| Likes about the program | Things that staff and patient like about the program, benefits or aspects that they like. |
| Promotion of the program | Promotion and awareness of the program, awareness about the program in the physio department, outside of the physio department, the public, GPs, patients, surgeons. Suggestions for promotion. May double code suggestions for promotional resources here and in the resources. |
| Strengths and Limitations of the GLA:D^®^ program | So any staff and patient descriptions about strengths and limitations of the program for example a strength may be the structure of the program and the timeframe for delivery and a limitation may be lack of screening tools and resources. |
| Ideas | Any ideas or suggestions made by the interviewees about the program or OA management |
| Managing OA | Management of OA, by clinicians or patients |
| Memorable quotes | Any quotes that we want to remember, think are significant or describe a concept clearly and might be useful for publication, the report or analysis. Any controversial quotes that require discussion can be coded here. |
| Multilevel support | any references (participants' comments) about the importance of internal and external support (local leaders /regional and national level support) to implement GLA:D^®^ in their settings. |
| Patients | Any reference by staff and patients’ interviewees about patients are coded here. This includes staff perception about patients, any references made and any more specific references to outcomes, responses and self-management are coded to the specific daughter nodes. |
| Diagnosis and Previous management | Any references mention previous health professional consultation about patients' OA and advice or recommendation about GLA:D^®^ and exercise, education, and surgery before participating in the GLA:D^®^ program. |
| Enrol in the GLA:D^®^ program | Any reference about how patients heard about the GLA:D^®^ and came to participate in the program. |
| Patient Outcomes | Any references to patient outcomes, including changes in pain, changes in function, improvement in pain, changes in their management plan, changes in medication, Self-Management and Adherence (completing GLA:D^®^ or dropping out), adherence to the at-home exercise program (including whether or not patients are doing the exercises at home), adherence to the exercises and continuing exercises or activity after the program has ceased. Any references made by staff about self-management and patient's ability to self |
| Patients' experience in the GLA:D^®^ program | Any comments about how the patients experience the program. |
| Referral Pathway | References to referral for GLA:D^®^, decision making about who is referred for GLA:D^®^, discussion about mode of referral (self-referral, GP, surgeon or orthopaedics). Referral processes including efficiency, complexity of the referral process, any potential improvements to the referral process. |
| Waiting List | Any references to the waiting list, management of the waiting list for GLA:D^®^, time patients are on the waiting list. And priority of patients on the waiting list for GLA:D^®^ not being a high priority due to their overall low priority status. OA being low priority within the physio service due to severity/or any other reason. |
| Resources | Potential resources, any references to existing resources or the requirement for more. For example: the existence of the role’s manual, the need for a screening tool. Any suggestions about promotional material or educational resources for patients or staff. |
| Role | Interviewee description of their role, within the THS and as part of GLA:D^®^. Experience, duties, qualifications, GLA:D^®^ & THS). Reference to roles throughout the interview. |
| Administrative | Any reference to administrative roles, code all interviews with administrative staff here too. |
| Allied Health Assistant | All allied health assistant roles and duties are coded here, as well as interviews with allied health assistants. |
| Clinical | All references to clinical roles are coded here. |
| Sustainability | Comments or references about sustainability of the program, anything that would be required for the program to continue, we code here whether or not staff think the program should continue, anything that would need to change for the program to continue |
| Telehealth | All mentions of telehealth are coded here including descriptions of potential telehealth models. And any current or previous use of telehealth in GLA:D® in the THS. Any ideas about future use of telehealth. |
| Understanding of GLA:D^®^ | Interviewee descriptions of what they understand GLA:D^®^ to be about, describing the program, definition of GLA:D^®^. |
